# A report of three cancer patients on opioid analgesia receiving spinal anesthesia: abrupt pain elimination without respiratory depression

**DOI:** 10.1186/s40981-020-00355-2

**Published:** 2020-07-01

**Authors:** Natsuko Nozaki-Taguchi, Yuko Ueda, Azusa Inada, Kyongsuk Son, Shiroh Isono

**Affiliations:** 1 Department of Anesthesiology, Graduate School of Medicine, Chiba University, 1-8-1 Inohana, Chuo Chiba, 260-8670 Japan; 2 Department of Anesthesiology, Chiba Cerebral and Cardiovascular Center, Chiba, Japan; 3 Department of Anesthesiology, Chiba Emergency Medical Center, Chiba, Japan; 4 Department of Anesthesiology, Chiba University Hospital, Chiba, Japan

**Keywords:** Chronic opioid, Respiratory depression, Tolerance

## Abstract

**Background:**

Complete removal of pain with regional anesthesia has been reported to cause fatal respiratory depression in opioid-dependent patients, which leads us to choose general anesthesia. We hereby report three cases of chronically opioid-treated cancer patients operated under spinal anesthesia without respiratory event.

**Case presentation:**

Case 1: a 32-year-old female treated with high-dose morphine for her cancer pain was planned for cesarean section. Case 2: a 65-year-old female on moderate dose of oxycodone was planned for surgery of her femoral bone fracture. Case 3: a 65-year-old male on low-dose oxycodone was planned for intramedullary nailing for metastatic femoral bone tumor. In all three cases, spinal anesthesia was chosen. Continuous respiratory monitoring revealed no apnea or bradypnea.

**Conclusion:**

Spinal anesthesia was safely performed without respiratory depression in chronic opioid users for cancer pain.

## Background

Recently, the number of patients coming to the operating room with opioid analgesics is increasing, as opioids are used more regularly in both cancer and non-cancer pain. It is often believed that sudden and complete removal of pain with neuraxial anesthesia causes respiratory depression in opioid-consuming patients, since pain itself is considered to antagonize the respiratory depressant effect of opioids [1]. Case reports show fatal respiratory distress [2, 3], which guides our decision to choose general anesthesia for intraoperative safety. Patient conditions may not allow general anesthesia, or in some cases, regional anesthesia is favored. We report here three cases of cancer pain patients with several dose range of opioid analgesia, successfully treated with regional anesthesia which eliminated both cancer and surgical pain without any respiratory event.

Written informed consent for case report was obtained from patient herself in case 1, and from family members of the deceased in cases 2 and 3. Institutional IRB approval for submission was gained in October 2019 (#3564).

This manuscript adheres to the applicable EQUATOR guideline.

## Case presentation

**Fig. 1:**
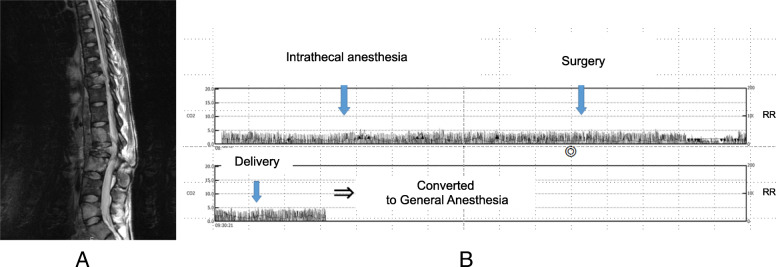
**a** Magnetic resonance imaging of the spine taken 8 months before the surgery showing multiple bone metastasis of the rectal cancer. **b** Compressed figure of capnography monitored during the surgery. Left and right axes show end-tidal CO_2_ and respiratory rate, respectively

Case 1: a 32-year-old pregnant woman (height 163 cm, body weight (BW) on admission 37 kg) was diagnosed with rectal cancer with liver and bone metastasis at 24 gestational weeks. Cancer pain due to bone metastasis of T12, L3 spine (Fig. 1a) was treated with morphine which dose increased gradually due to disease progression. She received chemotherapy until 32 gestational weeks when cesarean section was planned. At the time of surgery, after 8 weeks opioid use, she was suffering from severe low back pain and lancinating pain in her semi-paralyzed left leg. She was treated with oral paracetamol and intravenous morphine 60 mg/h with frequent rescue doses, which totaled to as much as 2 g/day. Since the baby was planned to enter NICU while the patient was completely bedridden, the operating room could be her only chance to meet the baby. Regional anesthesia was chosen after careful assessment of the feasibility, risk, and the mother’s will. Anesthesia was induced with 2.4 ml hyperbaric 0.5% bupivacaine which produced anesthesia up to T9 dermatome, which also treated her cancer pain completely for few hours. Intraoperative monitoring consisted of ECG, SpO_2_, and non-invasive blood pressure (BP) measurement. Respiratory rate (RR) and end-tidal CO_2_ were continuously monitored with capnography (WEC-7301: NIHON KOHDEN, Japan) attached to a nasal cannula supplying 2 L/min oxygen during the surgery. Capnography revealed no apnea nor bradypnea (Fig. 1b). Average RR during surgery was 18 ± 7/min, and lowest RR observed was 9/min. After the mother had met the baby, she was given general anesthesia while colostomy was performed (total surgery time: 1 h 16 min). After recovery from general anesthesia, still with anesthesia level to T10, continuous respiratory monitoring was applied for 6 h which revealed average RR of 20 ± 8/min with no apnea. In short, her cancer pain in addition to postoperative pain returned.

**Fig. 2:**
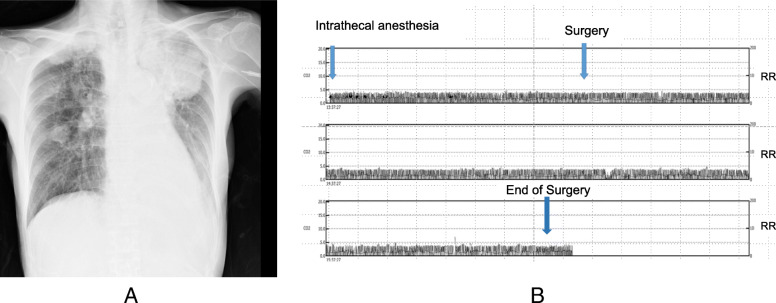
**a** Preoperative chest x-ray of the patient showing lung metastatic tumor compressing the trachea forming atelectasis of the left upper lobe. **b** Compressed figure of capnography monitored during the surgery. Left and right axes show end-tidal CO_2_ and respiratory rate, respectively

Case 2: a 65-year-old female (153 cm, 39 kg) was diagnosed with recurrence of esophageal cancer and metastasis of lung and bone. She was receiving intravenous oxycodone which was gradually increased for 18 days to 4.5 mg/h for her pain due to metastasis of her right sacral bone. Femoral trochanteric fracture was diagnosed after tumbling on the floor and she was planned for intramedullary nailing. As her preoperative chest x-ray revealed narrowing of the trachea due to lung metastasis (Fig. 2a), regional anesthesia with spinal anesthesia was selected. Isobaric 0.5% bupivacaine 2 ml completely treated her pain due to both fracture and bone metastasis (no record of anesthesia level). Oxycodone was continued during the surgery (surgery time, 1 h 48 min) and continuous RR and end-tidal CO_2_ monitoring was applied in accordance with the standard monitoring of ECG, SpO_2_, and BP. No respiratory event was recorded throughout the surgery (Fig. 2b) with average RR of 18 ± 6/min.

**Fig. 3:**
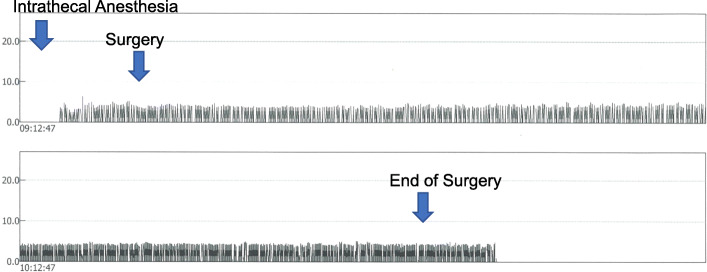
Compressed figure of capnography monitored during the surgery. Left and right axes show end-tidal CO_2_ and respiratory rate, respectively

Case 3: a 65-year-old male (182 cm, 67 kg) diagnosed with femoral metastasis of gastric cancer was planned for elective intramedullary nailing. He was receiving oral oxycodone 20 mg/day for more than 50 days. His bone cancer pain was under control. Regional anesthesia was chosen as the surgery was a simple procedure. He took the regular morning dose of oxycodone before surgery and lumbar anesthesia was performed with 2.6 ml hyperbaric 0.5% bupivacaine, and he was free from pain (no record of anesthesia level). Continuous RR and end-tidal CO_2_ monitoring with the standard monitoring during the surgery showed no apnea nor bradypnea during the surgery (surgery time, 1 h 33 min) (Fig. 3) with average RR of 18 ± 5/min while he was observed to be sleeping without any sedatives during the operation.

## Discussion

Analgesic effect of opioid, the major drug in pain treatment, has always been balanced with its side effect, especially respiratory depression. Existence of pain is known to stimulate respiration, thus antagonizes the respiratory depressant effect of opioids [1]. Abrupt removal of the pain by local anesthetics changes this balance between pain and respiratory depression. Combes et al. [4] have shown that abolition of postoperative pain by regional anesthesia in opioid-treated patients increased the incidence of abnormal respiratory events associated with oxygen desaturation.

In the case of chronic opioid users, the problem becomes more complex. In patients with long-time opioids, they are likely to have developed tolerance to both the analgesic effect and also the respiratory depressant effects. However, the level of tolerance may vary. The level of tolerance depends, possibly on the total dose or duration of opioids, but we have no clinically measurable method.

Few case reports show us the risk or respiratory arrest after complete and sudden abolition of pain by regional anesthesia [2, 3]. Our report is the first to show result from continuous monitoring on respiration under these conditions. Whether respiratory arrest can be predicted by slowing of respiratory rate or increase in irregular breathing is not well investigated. In our three cases, opioid dose ranged from small dose of 20 mg of po oxycodone to 2 g of iv morphine. Duration of opioid use ranged from 18 days to 2 months. Tolerance level to opioid is probably different amongst three patients and also amongst opioid effect, analgesia, and respiratory depression. We observed no bradypnea, no apnea, and not even decreased respiratory rate in all three patients after removal of pain. They seemed to be totally tolerant to the respiratory depressant effect of opioids at doses given preoperatively. The observation of increased respiratory events treated with opioids in opioid naïve patients [4], compared with our results suggest us that tolerance to respiratory depression may develop within weeks. However, this needs further investigation.

Our clinical observation of no respiratory depressant events shows that, under continuous and intent monitoring, choice of regional anesthesia in chronic opioid-consuming patients with clear indication for neuraxial block can be safely managed. Especially in long-time opioid users, we experience the need for escalated dose of postoperative opioid [5] or even intraoperative opioid, which sometimes results in opioid-induced hyperalgesia postoperatively and uncontrolled pain after surgery [6]. Regional anesthesia may be a better choice in such patients. Though we do not have clear evidence, general anesthesia is considered to produce immunosuppression such may affect malignant tumor recurrence [7], another reason to prefer regional to general anesthesia. Importantly, unexpected fatal respiratory arrest being reported after regional anesthesia in long-term opioid consumers [2, 3], continuous monitoring of respiration is mandatory under these conditions. Bradypnea is often not accompanied with desaturation with supplemental oxygen. Accordingly, oximetry may be a late indicator for hypoventilation. Though capnometer may not be an accurate end-tidal CO_2_ monitor when used in spontaneously breathing patients, it is strongly recommended for early detection of apnea or bradypnea [8].

## Conclusion

In conclusion, regional anesthesia which may completely abolish the cancer pain in patients under chronic opioid analgesia can be safely administered. As presence or level of tolerance to respiratory depressant effect of opioids cannot be detected preoperatively, continuous monitoring is mandatory.

## Data Availability

Data sharing is not applicable to this article as no datasets were generated or analyzed during the current study.

## Abbreviations

RR: Respiratory rate
BP: Blood pressure
